# Could anterior closed-wedge high tibial osteotomy be a viable option in patients with high posterior tibial slope who undergo anterior cruciate ligament reconstruction? A systematic review and meta-analysis

**DOI:** 10.1007/s00590-022-03419-4

**Published:** 2022-10-29

**Authors:** Francesco Bosco, Fortunato Giustra, Riccardo Giai Via, Alessandro Dario Lavia, Marcello Capella, Luigi Sabatini, Salvatore Risitano, Giorgio Cacciola, Daniele Vezza, Alessandro Massè

**Affiliations:** 1 Department of Orthopaedics and Traumatology, University of Turin, CTO, Via Zuretti 29, 10126 Turin, Italy; 2 Department of Economics, Boston College, Boston, MA USA

**Keywords:** ACL, Anterior cruciate ligament, ACLR, ACL reconstruction, Anterior closed-wedge high tibial osteotomy, Ligament reconstruction, Posterior tibial slope, PTS, Knee stability, Graft insufficiency, Graft failure, Outcomes, Tibial deflexion osteotomy, Revision surgery

## Abstract

**Purpose:**

This study aims to examine the clinical and radiological outcomes of patients who underwent ACL reconstruction (ACLR) combined with anterior closed-wedge high tibial osteotomy (ACW-HTO) for posterior tibial slope (PTS) reduction to investigate the efficacy of this procedure in improving anterior knee stability and preventing graft failure in primary and revision ACLR.

**Methods:**

A literature search was conducted in six databases (PubMed, Embase, Medline, Web of Science, Cochrane, and Scopus). The study was performed according to the Preferred Reporting Items for Systematic Reviews and Meta-Analyses (PRISMA) guideline. The initial screening identified 1246 studies. Each eligible clinical article was screened according to the Oxford Centre for Evidence-Based Medicine 2011 levels of evidence (LoE), excluding clinical studies of LoE V. Quality assessment of the articles was performed using the ROBINS-I methodological evaluation. This systematic review and meta-analysis was registered on the International Prospective Register of Systematic Reviews (PROSPERO). For the outcomes that were possible to perform a meta-analysis, a *p* < 0.05 was considered statistically significant.

**Results:**

Five clinical studies were included in the final analysis. A total of 110 patients were examined. Pre- and post-operative clinical and objective tests that assess anteroposterior knee stability, PTS, clinical scores, and data on surgical characteristics, complications, return to sports activity, and graft failure after ACLR were investigated. A meta-analysis was conducted using R software, version 4.1.3 (2022, R Core Team), for Lysholm score and PTS outcomes. A statistically significant improvement for both these clinical and radiological outcomes (*p* < 0.05) after the ACW-HTO surgical procedure was found.

**Conclusion:**

ACLR combined with ACW-HTO restores knee stability and function with satisfactory clinical and radiological outcomes in patients with an anterior cruciate ligament injury associated with a high PTS and seems to have a protective effect from further ruptures on the reconstructed ACL.

**Level of evidence:**

Level IV.

## Introduction

Anterior cruciate ligament (ACL) injuries are relatively common, with an average incidence of 29–38 per 100,000 inhabitants [1–3]. They mainly affect the young and athletic population, but in recent decades, ACL injuries have also been reported in adults and paediatric patients [4]. ACL reconstruction (ACLR) is one of the most widely performed surgical procedures in orthopaedics, with good results in patient satisfaction and high rates of return to previous sports activity; nevertheless, treatment failure rates range from 10 to 20 per cent [5].

Several risk factors potentially responsible for ACLR failure have been analysed and classified into intrinsic and extrinsic [6]. Historically, great emphasis has been placed on extrinsic factors, such as graft choice, diameter, and tensioning or tunnel placement and reconstruction technique, with gradual and continuous progress leading to improvements in ACLR outcomes [7–9]. In recent years, more attention has been directed to intrinsic factors, especially metaphyseal coronal malalignment, and posterior tibial slope (PTS), that had rarely been considered and corrected simultaneously with ACLR [10, 11]. Malalignment in the coronal plane may cause an alteration in loading between the medial and lateral compartments, resulting in an increased risk of meniscal and cartilage damage and faster progression of osteoarthritis in the compartment with higher loading [12–14]. The increased PTS may be responsible for reduced knee stability after ACLR [11]. A high PTS results in greater anteriorly directed shear forces on the ACL with an excessive anterior tibial subluxation in extension. In contrast, a flatter PTS reduces the tensile forces on the ACL by increasing the load on the posterior cruciate ligament (PCL) [11, 15, 16]. According to these biomechanical studies, many authors suggested that patients with ACL rupture and PTS values greater than 12° may benefit from a combined ACLR and anterior closed-wedge high tibial osteotomy (ACW-HTO) [11, 14, 15]. ACLR improves knee biomechanics by correcting anteroposterior instability, while ACW-HTO may have a protective effect on ligamentous reconstruction by reducing shear forces on the neo-ACL [17].

This systematic review and meta-analysis aims to investigate the clinical and radiological outcomes of patients who underwent ACW-HTO for slope reduction simultaneously with ACLR to investigate the efficacy of this procedure in improving anterior knee stability and preventing graft failure in primary and revision ACLR.

## Materials and methods

### Research question

**Fig. 1:**
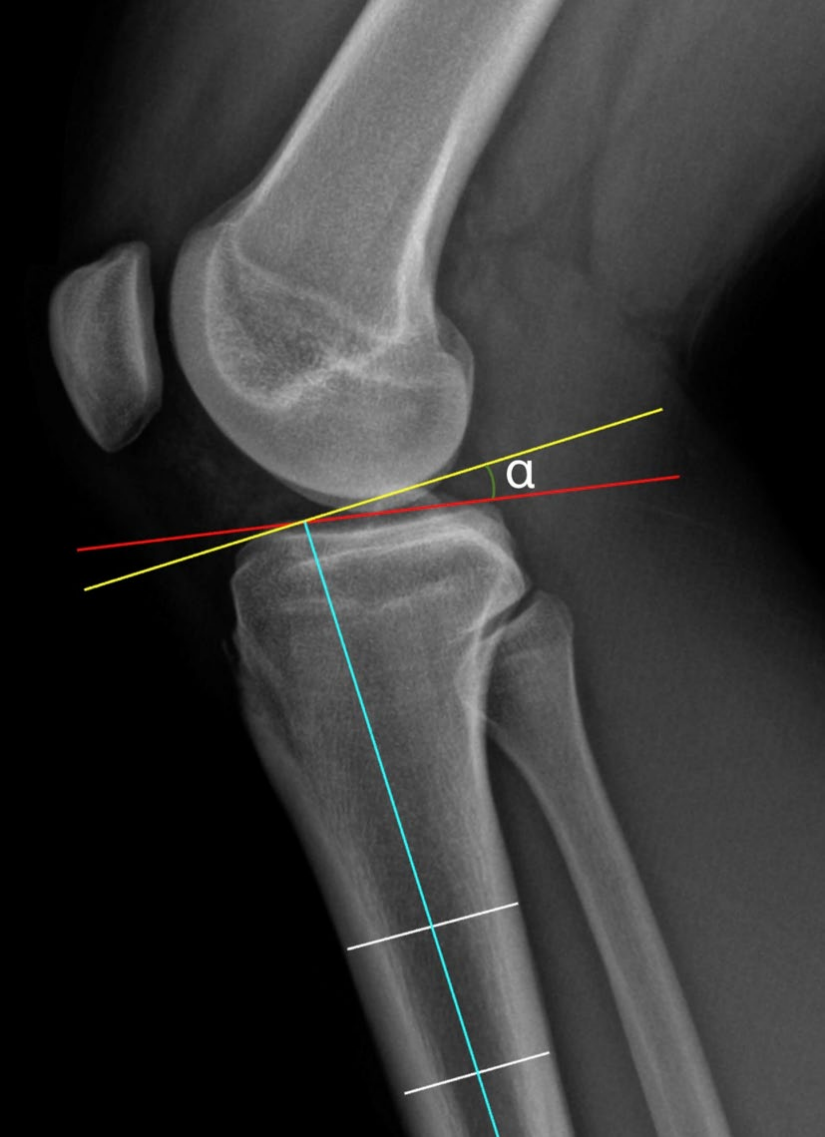
True lateral’ radiograph of a knee. The posterior tibial slope (PTS) is the angle (*α*) between the perpendicular (yellow line) to the tibial longitudinal axis (light blue line) and the tangent to the anterior and posterior edges of the medial tibial plateau (red line) as described by Dejour et al. [11] (colour figure online)

A systematic literature review was conducted to evaluate studies that analysed clinical and radiographic outcomes of patients who underwent an anterior closed-wedge high tibial osteotomy (ACW-HTO) to reduce the high PTS (Fig. 1) concomitantly with or before ACLR. The current study was conducted following the Preferred Reporting Items for Systematic Reviews and Meta-Analyses (PRISMA) [18]. Two authors (FB and RGV) searched and evaluated the articles independently to avoid possible bias. A third author (FG) was consulted to resolve any doubts.

### Search strategy and study screening

**Fig. 2:**
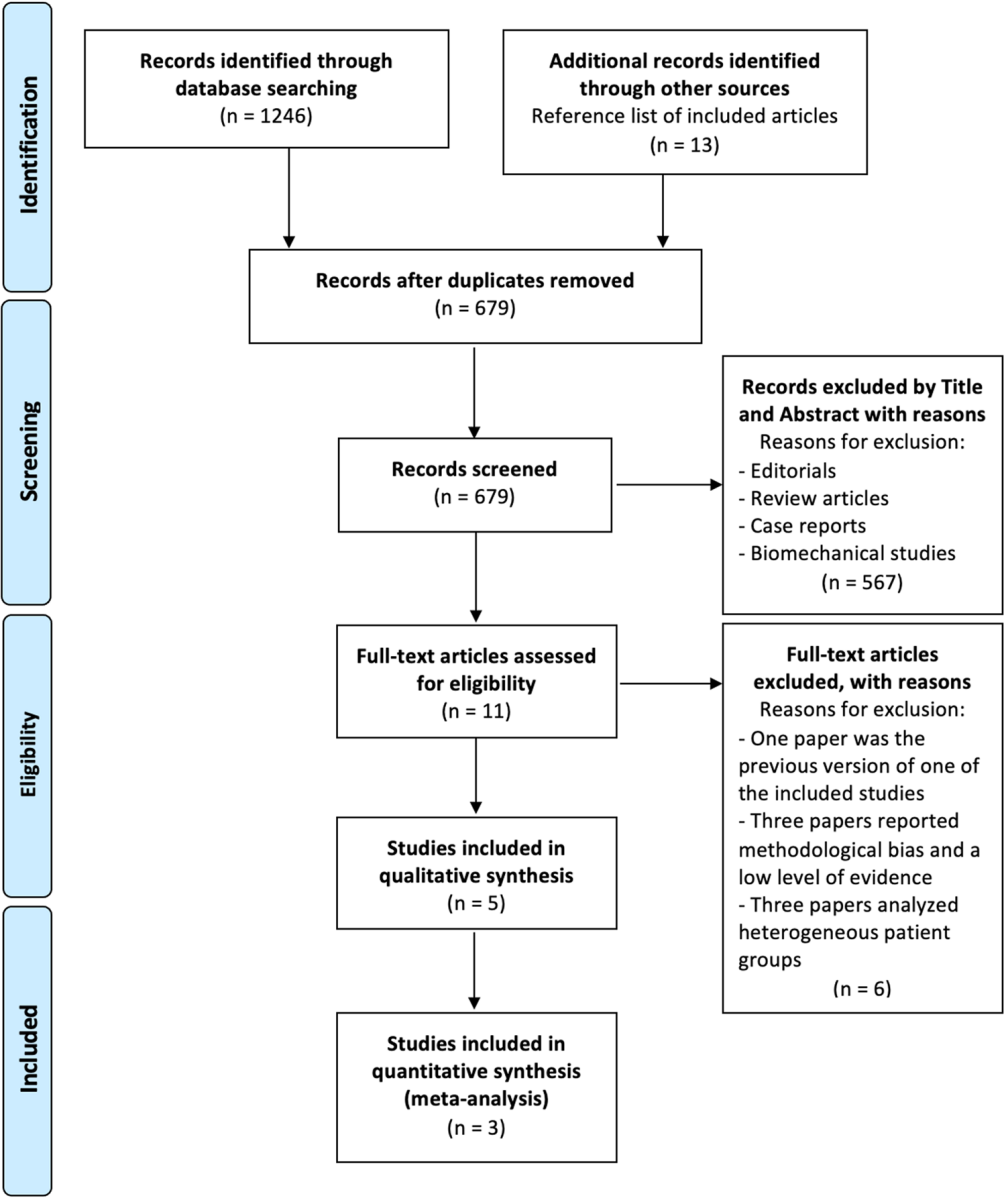
Preferred reporting items for systematic reviews and meta-analyses (PRISMA). Flow diagram of articles included in this systematic review and meta-analysis

A literature search was performed in six databases (Pubmed, Embase, Medline, Web of Science, Cochrane, and Scopus) using the following terms: [(sagittal tibial osteotomy) OR (deviation osteotomy) OR (slope reduction tibial osteotomy) OR (tibial slope)] And [(anterior cruciate ligament) OR (ACL) OR (ACLR) OR (anterior cruciate ligament reconstruction) OR (anterior cruciate ligament revision)]. The search included studies from January 2000 to August 2022. A total of 1246 studies were identified. After the exclusion of duplicates, 679 studies were included. After title and abstract screening, eleven clinical studies were assessed for full-text evaluation, and five clinical studies [15, 17, 19–21] were finally included in this systematic review based on the inclusion and exclusion criteria. A cross-check was performed for additional studies to be included in the current study. The PRISMA flowchart for study selection is shown in Fig. 2 [18].

### Inclusion and exclusion criteria

The inclusion criteria were studies that included patients who underwent ACW-HTO as a complement to anterior cruciate ligament reconstruction, written in English, studying human subjects, published between January 2000 and July 2022 with a minimum follow-up of six months, RCTs, prospective and retrospective studies with Oxford Centre for Evidence-Based Medicine 2011 Levels of Evidence (LoE) 1–4 [22]. Biochemical and in vitro studies, case reports, preclinical studies, editorials, book chapters, technical reports, and review articles were excluded from the search. We also excluded studies that analysed patients treated with both coronal and sagittal tibial osteotomy and studies with LoE 5 for better quality studies.

### Quality assessment

**Fig. 3:**
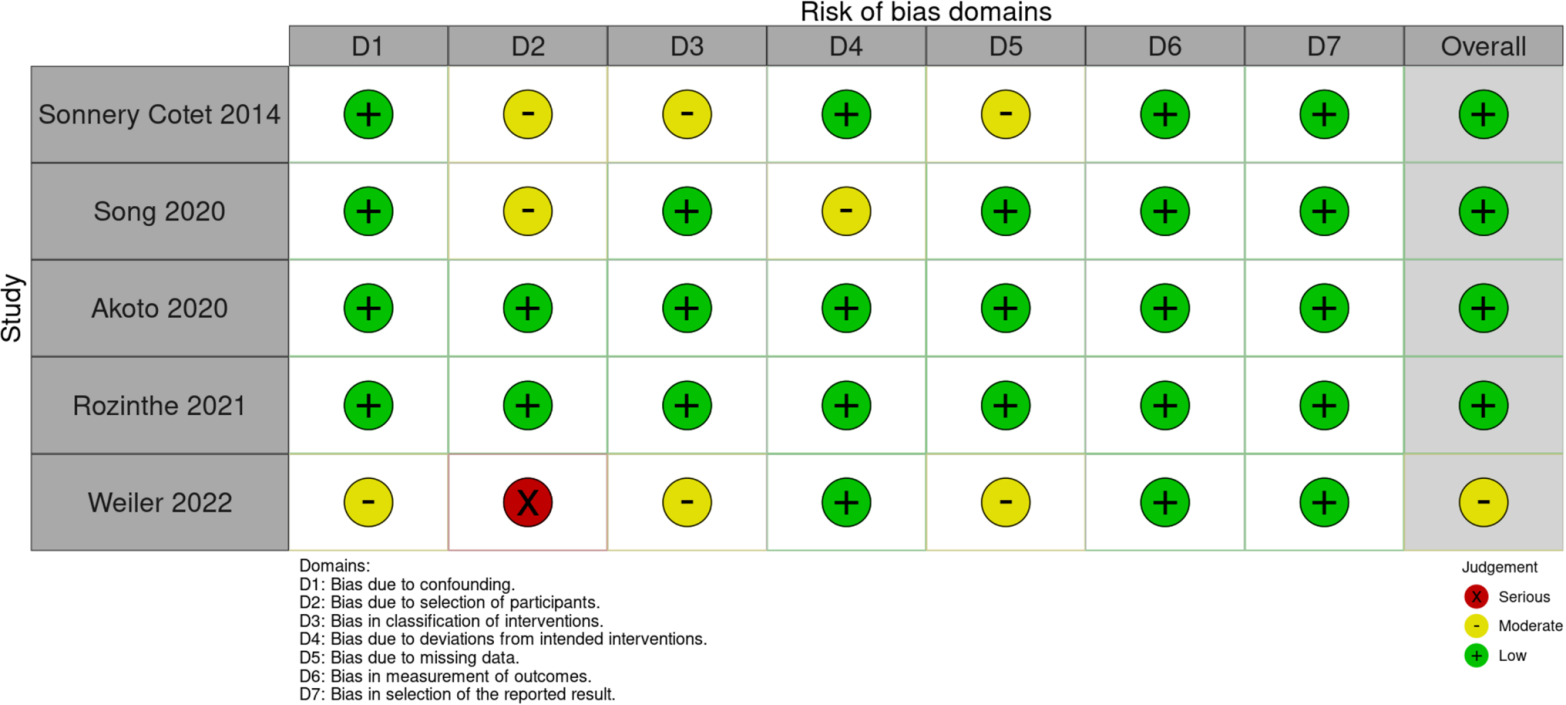
Risk of bias in non-randomized studies—of interventions (ROBINS-I) tool assessment. Risk of bias conformed by the Cochrane Handbook for Systematic Reviews of Interventions. The quality and risk of bias of individual retrospective studies included in the systematic review

Each article included in this systematic review was examined following the LoE [22]. A Risk of Bias In Non-randomized Studies—of Interventions (ROBINS-I) [23, 24] was used to analyse the included studies (Fig. 3). This tool was used by two authors (RGV and FB), and a third author (FG) was employed to support resolving any additional uncertainties. Statistical analysis was performed by a professional statistician (LDA). Study design, manuscript writing, and final editing were equally distributed among the authors. This systematic review was registered in the International Prospective Register of Systematic Reviews (PROSPERO), CDR CRD42022333255, in May 2022 [25–27].

### Data extraction

Data extracted from included articles were reported on a template: authors and publication; study design; LoE; sample size of patients; sample size of mean age; sample size of sex; study follow-up; patients lost to follow-up; pivot-shift test; objective side-to-side differential anterior laxity; type of tendon graft used for anterior cruciate ligament revision; stage procedure/primary-revision ACLR; surgical technique used to perform the ACW-HTO; additional surgical meniscal treatments; complications and graft failure after ACLR; PTS; subjective and objective pre and post-operative clinical scores, and sample size of patients that return to sport.

### Data analysis

Lysholm score and PTS have been considered for a meta-analysis since they were present in two and three studies analysed [17, 19, 21] allowing for a valid statistical comparison. The analysis has combined the data as standardised mean differences (SMD), using random-effect analysis and inverse weighting for pooling. The average effect size and a 95% confidence interval have been computed via the Jackson method. Cochran’s *Q* test and Higgins’ *I*^2^ statistics have been performed to check for heterogeneity between studies. The SMD requires a *p* value of 0.05 to be considered statistically significant. Funnel plots and Egger’s tests have been performed to test for eventual publication bias. The statistical analysis was performed using R software, version 4.1.3 (2022, R Core Team).

## Results

A total of 110 patients were analysed in this study. The main demographic characteristics such as age, mean follow-up, number and percentage of males and females are summarised in Table 1. Pre- and post-operative clinical and objective tests to assess anteroposterior knee stability are recorded in Table 2. Surgical techniques, the number of surgery stages and any associated meniscal procedures performed by the several authors of the included studies are reported in Table 3. Pre-operative and post-operative values of PTS, clinical scores, and return to sport are shown in Table 4. Finally, complications and graft failure after ACLR are reported in Table 5.

**Table 1:** Main demographic characteristics of patients collected in studies included in this systematic review and meta-analysis

Author and publication year	Study design	LoE	Sample size patients, initial cohort/final cohort	Age	*M*	*F*	Follow-up	Patient lost to follow-up
			*N*	Mean ± SD/(range), y.o	*N* (%)	*N* (%)	Mean ± SD/(range)	*N*
Sonnery-Cottet et al. 2014 [20]	RS	IV	5/5	24 (16–40)	4 (80%)	1 (20%)	31.6 (23–45) months	0
Song et al. 2020 [15]	RS	IV	18/18	29.4 (20–41)	16 (89%)	2 (11%)	33.2 (25–44) months	0
Akoto et al. 2020 [19]	RS	IV	22/20	27.8 ± 8.6	14 (70%)	6 (30%)	30.5 ± 9.3 (24–56) months	2
Rozinthe et al. 2021 [17]	RS	IV	9/8	30.3 ± 4.4 (21–49)	5 (66.67)	3 (33.3)	9.9 ± 3 (7–15) years	1
Weiler et al. 2022 [21]	RS	IV	58/58	32.2*	47 (62%)*	29 (38%)*	Minimum: 6 months**	0

LoE: Oxford Centre for Evidence-Based Medicine 2011 Levels of Evidence; M—male; F—female; N—number of evaluation cases; y.o.—years old; SD— standard deviation; %—percentage; RS—retrospective study; *: Demographic data of entire study patients are reported, including patients who underwent sagittal correction (ACW-HTO) or a combined procedure with an additional coronal realignment (medial open-wedge high tibial osteotomy (MOW-HTO)); **: Weiler A et al. 2022 [21] reported only the minimum follow-up

**Table 2:** Pre- and post-operative clinical and objective tests to assess anteroposterior knee stability

Author and publication year	Pivot-shift test								Side-to-side differential anterior laxity	
	Pre-op				Post-op				Pre-op	Post-op
	0	1 +	2 +	3 +	0	1 +	2 +	3 +	Mean ± SD/(range), mm	Mean ± SD/(range), mm
Sonnery-Cottet et al. 2014 [20]	0	1	3	1	4	1	0	0	10.4 (8–14)*	2.8 (2–4)*
Song et al. 2020 [15]	0	0	15	3	18	0	0	0	13 (10–15)**	1.6 (− 4 to 3)**
Akoto et al. 2020 [19]	0	0	0	20	20	0	0	0	7.2 ± 1.3***	1.1 ± 1.1***
Rozinthe et al. 2021 [17]	N/A	N/A	N/A	N/A	N/A	N/A	N/A	N/A	N/A	N/A
Weiler et al. 2022 [21]	N/A	N/A	N/A	N/A	N/A	N/A	N/A	N/A	N/A	N/A

Pre-op—pre-operative; Post-op—post-operative; SD—standard deviation; mm—millimetre; N/A—not specified; *The side-to-side differential anterior laxity was evaluated with the Telos device; **The side-to-side differential anterior laxity was evaluated with the KT-1000 arthrometer; ***The side-to-side differential anterior laxity was evaluated with the Rolimeter test

**Table 3:** Surgical characteristics

Author and publication year	Meniscectomies or meniscal sutures (medial meniscus status)			Meniscectomies or meniscal sutures (lateral meniscus status)			ACLR graft			Stage procedure /P-R ACLR	Osteotomy surgical technique
	Prior to ACW-HTO	At ACW-HTO	After ACW-HTO	Prior to ACW-HTO	At ACW-HTO	After ACW-HTO	QT	HT	BPTB		
	*N*	*N*	*N*	*N*	*N*	*N*	*N*	*N*	*N*		
Sonnery-Cottet et al. 2014 [20]	1	0	N/A	2	2	N/A	4	0	1	One stage/R	ACW-HTO with detachment of ATT and patellar tendon
Song et al. 2020 [15]	N/A	17	N/A	N/A	8	N/A	0	18	0	One stage/P	ACW-HTO without detachment of ATT and patellar tendon
Akoto et al. 2020 [19]	N/A	N/A	12	N/A	N/A	0	12	7	1	Two stages/R	ACW-HTO with detachment of ATT and patellar tendon
Rozinthe et al. 2021 [17]	6	4	N/A	1	7	N/A	8	1	0	One stage/R	ACW-HTO without detachment of ATT and patellar tendon
Weiler et al. 2022 [21]	N/A	N/A	N/A	N/A	N/A	N/A	N/A	N/A	N/A	Two stages/P	ACW-HTO with or without detachment of ATT and patellar tendon

ACLR—anterior cruciate ligament reconstruction; P—primary ACLR; R—revision ACLR; ACW-HTO—anterior closed-wedge high tibial osteotomy; QT—quadriceps tendon; HT—hamstring tendon; BPTB—bone-patellar tendon-bone; ATT—anterior tibial tuberosity; *N*—number of evaluation cases; N/A—not specified; One stage: slope-reducing tibial osteotomy combined with primary ACLR; Two stages: slope-reducing tibial osteotomy was performed first, and then revision ACLR

**Table 4:** Pre-operative and post-operative values of posterior tibial slope (PTS), clinical scores, and return to sport

Author and publication year	Posterior tibial slope		Lysholm score		Subjective IKDC questionnaire		Objective IKDC questionnaire*		Tegner activity score		Return to sport
	Pre-op	Post-op	Pre-op	Post-op	Pre-op	Post-op	Pre-op	Post-op	Pre-op	Post-op	
	Mean ± SD/(range)	Mean ± SD/(range)	Mean ± SD/(range)	Mean ± SD/(range)	Mean ± SD/(range)	Mean ± SD/(range)	Grade	Grade	Mean ± SD/(range)	Mean ± SD/(range)	*N* (%)
Sonnery-Cottet et al. 2014 [20]	13.6° (13°–14°)	9.2° (8°–10°)	46.2 (26–69)	87.8 (60–100)	39.5 (21.8–64–4)	79.1 (48.3–98.9)	3 C; 2 D	1 A; 4 B	7.4 (5–9)	7.2 (5–9)	4 (80%) **
Song et al. 2020 [15]	18.5° (17°–20°)	8.1° (7°–9°)	46.5 (34–58)	89.5 (78–94)	N/A	N/A	18 D	14 A; 4 B	5.7 (4–6)	7.3 (6–8)	18 (100%) **
Akoto et al. 2020 [19]	15.3° ± 11.6°	8.9° ± 11.1°	49.9 ± 21 (0–70)	90.9 ± 6.4 (76–100)	N/A	87.4 ± 5.9 (75.9–100)	N/A	N/A	2.9 ± 1.5 (0–5)	6.1 ± 0.9 (5–8)	13 (65%)
Rozinthe et al. 2021 [17]	13.2° ± 2.6° (10°–18°)	4.4° ± 2.3° (2°–8°)	38.4 ± 16.4 (24–80)	84.5 ± 11.9 (59–95)	44.1 ± 16.1 (23–75)	82.9 ± 12.1 (61–98)	N/A	N/A	N/A	N/A	N/A
Weiler et al. 2022 [21]	14.6° ± 2.3°	6.5° ± 1.9°	N/A	N/A	N/A	N/A	N/A	N/A	N/A	N/A	N/A

Objective International Knee Documentation Committee (IKDC); *: *A* = normal knee, *B* = nearly normal knee, *C* = slightly abnormal knee, *D* = abnormal knee; **: all the patients returned to the same activity level at the last follow-up; Pre-op—pre-operative; Post-op—post-operative; SD—standard deviation; *N*—number of evaluation cases; %—percentage N/A—not specified; °—degree

**Table 5:** Complications and graft failure after ACLR

Author and publication year	Complications		Graft failure after ACLR
	Intra-op	Post-op	
	*N* (%)	*N* (%)	*N*
Sonnery-Cottet et al. 2014 [20]	0	0	0
Song et al. 2020 [15]	0	0	0
Akoto et al. 2020 [19]	0	1 (5%): haematoma	0
Rozinthe et al. 2021 [17]	0	0	0
Weiler et al. 2022 [21]	0	1 (1.7%): implant infection	N/A

ACLR—anterior cruciate ligament reconstruction; Intra-op—intra-operative; Post-op—post-operative; *N*—number of evaluation cases; %—percentage; N/A—not specified

**Fig. 4:**
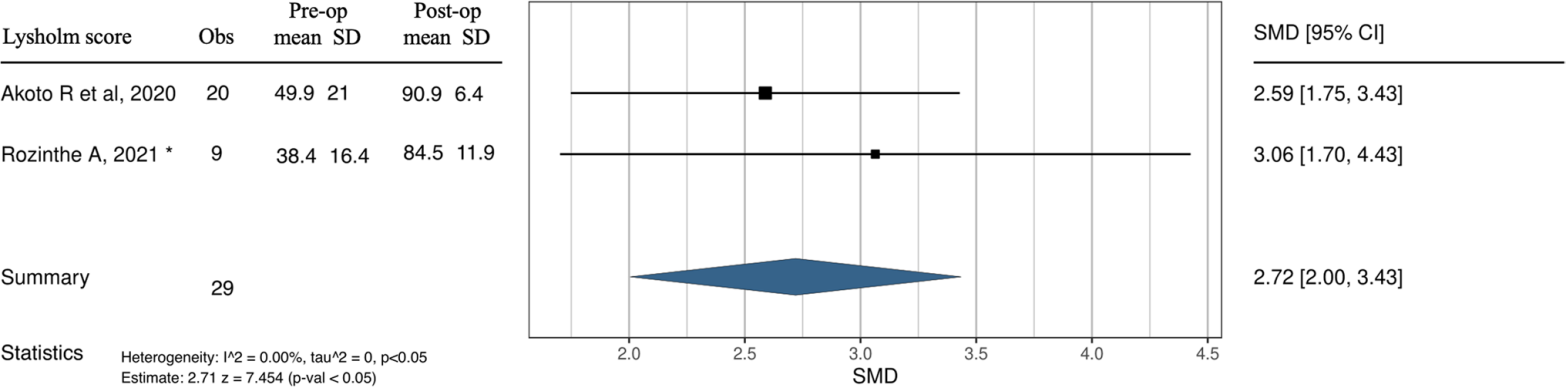
Forest plot. Comparison of Lysholm score results between pre-operative and post-operative. Obs—Observations; SD—standard deviation; SMD—standardised mean difference; CI—confidence interval; p—*p* value

**Fig. 5:**
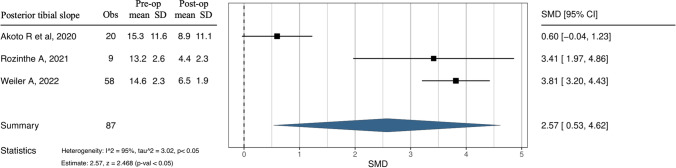
Forest plot. Comparison of posterior tibial slope degree values between pre-operative and post-operative. Obs—Observations; SD—standard deviation; SMD—standardised mean difference; CI—confidence interval; p—*p*. value

Therefore, 87 cases in three studies allowing for a valid statistical comparison were analysed [17, 19, 21]. Thus, it was possible to perform a meta-analysis of clinical outcome Lysholm score and PTS values difference pre-operatively and post-operatively. Both the forest and funnel plots show results in favour of no surgery on the left side for both outcomes considered for the quantitative analysis in this study. For the Lysholm score, as shown in Fig. 4, the Higgins statistic of 0% means no heterogeneity among the studies. The overall effect (2.71) is in favour of the surgical approach and is statistically significant (*p* < 0.05). The funnel plot indicates no publication bias, as shown in Appendix A (Egger's test). For PTS, measured in degrees, a favourable result is a value as close to zero as possible. As shown in Fig. 5, the Higgins statistic of 95% indicates moderate heterogeneity among the studies. The overall effect (2.57) is in favour of the surgical approach and is statistically significant (*p* < 0.05). The funnel plot reveals the potential presence of publication bias, as shown in Appendix B (Egger's test).

An interesting finding from the data analysis is a general tendency to encourage ACW-HTO and ACLR. All five studies included in this systematic review [15, 17, 19–21] reported an improvement in clinical performance, as demonstrated by the subjective and objective International Knee Documentation Committee (IKDC) and the Tegner activity scores (Table 4). Combined with the results of the meta-analysis, this evidence should suggest that in patients with high PTS requiring ACLR, there is a clinical advantage with the surgical approach of ACW-HTO.

## Discussion

The most important findings of this systematic review and meta-analysis were a statistically significant difference in the Lysholm score and PTS between the pre-operative and post-operative evaluation and the absence of ligamentous injuries or clinical knee instability during the entire follow-up period in patients who underwent ACW-HTO combined with ACLR. Furthermore, clinical scores like Tegner activity, subjective and objective IKDC analysed in the studies included in the systematic review and whose meta-analysis could not be performed were improved after surgical treatment [15, 17, 19–21]. These results demonstrate how this surgical procedure could restore good knee function and protect the ACLR in patients with anterior knee instability and high PTS.

ACLR is one of the most widespread orthopaedic procedures worldwide, with excellent clinical and functional results [3]. However, failure of primary ACLR and its subsequent revision is associated with inferior clinical scores compared to primary reconstruction [28–30]. Furthermore, several studies have demonstrated that multiple ACLR revisions could restore knee stability with good clinical and functional results, although inferior to previous reconstructions. However, only a small percentage of patients could return to their pre-injury activity level, with an overall higher rate of ACL failure [31, 32].

Several recent studies have investigated potential risk factors for ACLR failure to reduce ACL injury rates [28, 32, 33]. In line with improvements in surgical techniques, tunnel positioning, graft choice and fixation systems, the study of proximal tibia geometry, particularly the PTS, and how it may influence the biomechanics of the knee has become increasingly relevant. Agneskirchner et al., in their biomechanical study, demonstrated how an increase in PTS shifts the contact area of the tibial plateau anterosuperior to the femur, resulting in higher contact pressure on the anterior half of the tibial plateau in a linear relationship with increased PTS [34]. Dejour and Bonnin estimated that for every 10° increase in PTS, there is an anterior tibial translation relative to the femur of 6 mm in both intact and injured ACL [35]. Furthermore, a higher PTS results in a stronger traction force applied by the quadriceps during knee extension [1]. Biomechanical studies have demonstrated a linear relationship between a higher traction force applied to the ACL and an increase in PTS [33, 36]. Brandon et al. found a higher risk of pivot-shift and ACL rupture in patients with elevated PTS [10].

ACW-HTO and ACLR, in all studies analysed in this paper, proved to be surgical procedures characterised by good clinical and radiological results and a few complications in both primary reconstructions and multiple ACLR revisions. Sonnery-Cottet et al., in their study, reported a statistically significant improvement in the PTS, Lysholm, and subjective IKDC score and the reduction in mean anterior laxity, measured with the Telos device. An increase in the objective IKDC score and the pivot-shift test was also observed in the post-operative period. At the same time, no differences were reported in Tegner activity score. All but one patient returned to a level of sporting activity prior to the last ACL rupture, and no complications were observed in the follow-up [20]. Song et al. described a statistically significant improvement in PTS, Lysholm, Tegner activity and objective IKDC scores. The side-to-side difference measured with the KT1000 arthrometer and the pivot-shift test demonstrated a statistically significant increase. No complications occurred during the follow-up, and all patients returned to the same pre-injury sports activity level [15]. Akoto et al. reported statistically significant improvements in Lysholm and Tegner activity scores in their study. The PTS, visual analogue scale (VAS), and side-to-side differences measured with the Rolimeter and the pivot-shift test were statistically ameliorated significantly. Functional scores such as the Knee injury and Osteoarthritis Outcome Score (KOOS) and the subjective IKDC reported good results post-operatively. One patient underwent reoperation a few days after ACLR for a haematoma. Sixty-five per cent of the operated patients have returned to sports activities [19]. Rozinthe et al. updated the outcomes of patients undergoing ACW-HTO and ACLR, considering a minimum interval of seven years after surgery. The authors observed an improvement in Lysholm and subjective IKDC scores compared to the first evaluation. Lachman's and pivot-shift tests were negative in all patients, and no complications were observed during the follow-up. No PTS correction loss was reported compared to the previous follow-up [17]. Weiler et al., in their cohort study, analysed the change in PTS after surgery, describing a statistically significant decrease in PTS. One patient underwent implant removal due to infection approximately five months after surgery without any loss of reduction in the correction achieved [21].

Elevated PTS has been demonstrated to be an independent anatomical risk factor for excessive anterior tibial translation in the case of ACL injury [37]. Lee et al. found a significantly increased PTS in patients with ACLR failure compared to a control group with an uninjured ACL [38]. Webb et al., in their study, reported a five-fold increased probability of ACLR failure in patients with PTS ≥ 12° [39]. Grassi et al. suggested that an elevated PTS and an anterior tibial translation > 10 mm combination represents the situation with the highest risk of failure in ACLR. In particular, the authors underlined how excessive anterior tibial translation leads to an increased risk of tunnel malpositioning with a higher risk of neo-ACL impingement [40].

Biomechanical studies have demonstrated that ACW-HTO reduces the force on the ACL graft and decreases anterior tibial translation in the knee with ACL injury [33, 34, 36]. Nevertheless, the indication for ACW-HTO and ACLR is still debated in the literature. The main issues concern the correct PTS angle to be obtained and the use of ACW-HTO in primary or revision ACLR. Some authors have aimed for a PTS of 8°–10° [19, 20], while Rozinthe et al. [17] corrected the PTS to an average of 4°. While reducing PTS may improve anterior knee instability, it could also modify the proximal tibia geometry causing a change in the medial proximal tibial angle (MPTA) and leading to knee hyperextension [19, 21]. Weiler et al., in their study, reported a slight but significant inverse correlation between ACW-HTO width and changes in the coronal plane. Therefore, a higher sagittal plane correction is associated with a major risk of MPTA change [21].

Furthermore, PTS reduction may cause symptomatic genu recurvatum resulting in chronic pain and painful hyperextension of the knee during walking and standing [19]. In three of the five included studies, cases of knee hyperextension in the post-operative follow-up were reported, although all patients were asymptomatic [15, 17, 19]. The role of ACW-HTO and ACLR is greatly debated in primary or revision surgery. ACW-HTO is a technically demanding procedure associated with several complications, including popliteal bundle neurovascular lesions, tibial tubercle rupture and risk of pseudoarthrosis. In addition, this procedure increases the operative time and post-operative rehabilitation period [20]. For this reason, many authors consider ACW-HTO only in revision ACLR [17, 19, 20]. Instead, Song et al. and Weiler et al. performed ACW-HTO in primary ACLR in young, active patients with gross anterior instability and higher PTS, as they assumed that an ACLR alone could not restore proper knee biomechanics and stability. Furthermore, the authors emphasised that in experienced hands, ACW-HTO is an effective procedure with a low risk of complications [15, 21].

As reported in the included studies, the clinical outcomes of patients undergoing ACW-HTO and ACL reconstruction were similar to those described in other works in which only ACLRs were performed. A significant finding, also reported in the studies analysed in this systematic review and meta-analysis, is that ACLRs, particularly multiple revisions, were characterised by worse clinical outcomes than primary reconstructions [15, 17, 19–21, 28–30, 32].

This systematic review and meta-analysis is characterised by some limitations that need to be examined. Firstly, two included studies evaluate ACW-HTO and ACLR in primary ACLR; the other papers consider this surgical procedure in the ACLR revisions. A more homogeneous sample of patients could improve the validity of the analysed data. Secondly, different surgical techniques were used. Some authors performed ACW-HTO and ACLR in two stages [19, 21], others in one [15, 17, 20]. Furthermore, the osteotomy techniques proposed different management of the tibial tubercle and various osteotomy synthesis techniques. The absence of a standardised surgical procedure may lead to possible bias. Third, studies are few, retrospective, and with a limited sample of patients; this could potentially provide less statistical analysis. Moreover, follow-up periods are limited, except in the study by Rozinthe et al. [17]. Longer follow-ups with larger and more homogeneous samples may be needed to assess whether ACW-HTO and ACLR effectively prevent graft failure while ensuring good functional outcomes for treated patients. Fourthly, there is no standardised method to calculate PTS. In addition, some authors preferred short knee radiographs, whereas whole leg radiographs were considered in other studies. PTS values are influenced by the calculation method and the type of radiographs analysed, with a risk of potential bias.

The studies included in this systematic review and meta-analysis underline that high PTS is an aspect that should be evaluated in ACLRs because of its association with an increased risk of reconstruction failure. Furthermore, ACW-HTO and ACLR appear to be a surgical technique that, in the hands of experienced surgeons, could protect the ACL from subsequent rupture with clinical, functional, and radiographic results in line with isolated ACL reconstruction.

## Conclusion

This systematic review and meta-analysis reported a statistically significant difference in PTS and Lysholm scores associated with no ACL tears or knee instability in patients undergoing ACW-HTO and ACLR. Since high PTS values increased ACL anteriorly directed shear forces with a major risk of ACLR failure, the results reported in this article prove that ACW-HTO is a viable solution to restore knee stability and protect the ACLR in patients with anterior knee instability and high PTS.

## Figures and Tables

**Fig. 6:**
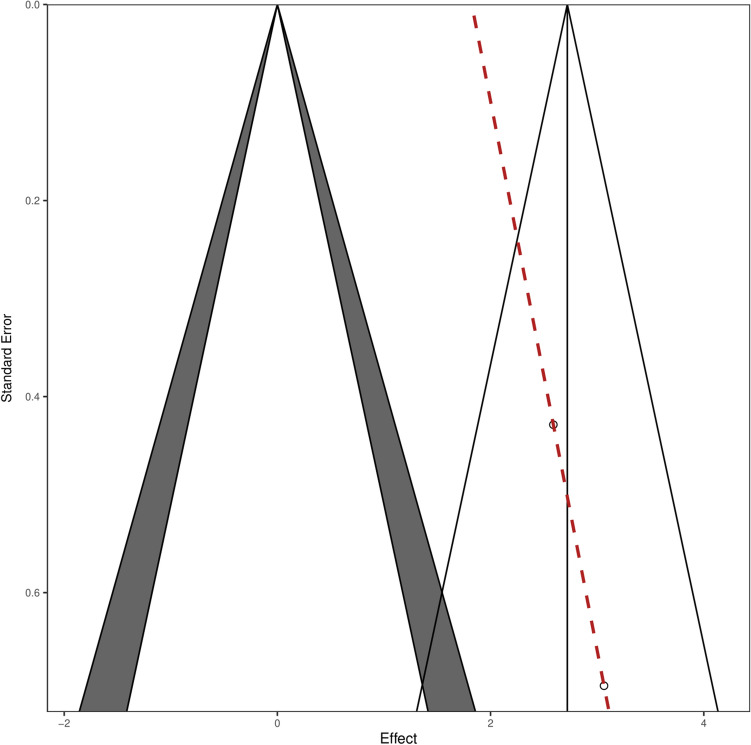
Funnel plot. Comparison of Lysholm score results between pre-operative and post-operative

**Fig. 7:**
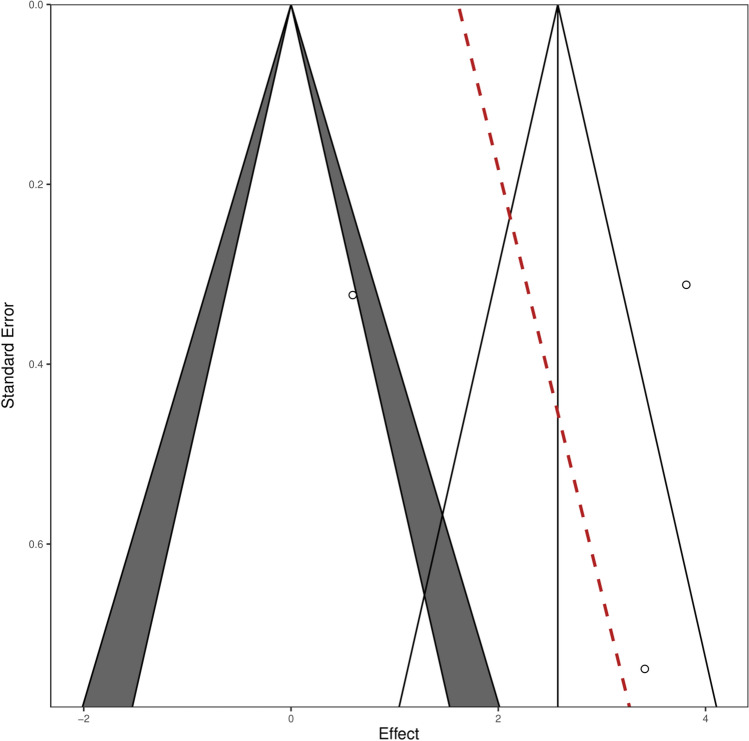
Funnel plot. Comparison of posterior tibial slope degree values between pre-operative and post-operative

## Appendix A

See Fig. 6.

## Appendix B

See Fig. 7.

## Abbreviations

ACL: Anterior cruciate ligament
ACLR: ACL reconstruction
PTS: Posterior tibial slope
PCL: Posterior cruciate ligament
ACW-HTO: Anterior closed-wedge high tibial osteotomy
PRISMA: Preferred Reporting Items for Systematic Reviews and Meta-Analyses
LoE: Oxford Centre for Evidence-Based Medicine 2011 Levels of Evidence
ROBINS-I: A Risk of Bias In Non-randomized Studies—of Interventions
PROSPERO: International Prospective Register of Systematic Reviews
SMD: Standardised mean differences
IKDC: International Knee Documentation Committee
VAS: Visual analogue scale
KOOS: Knee injury and Osteoarthritis Outcome Score
MPTA: Medial proximal tibial angle
